# Machine learning models predict the mTOR signal pathway-related signature in the gastric cancer involving 2063 samples of 7 centers

**DOI:** 10.18632/aging.204817

**Published:** 2023-06-20

**Authors:** Hao Zhang, Huiqin Zhuo, Jingjing Hou, Jianchun Cai

**Affiliations:** 1 Department of Gastrointestinal Surgery, Zhongshan Hospital of Xiamen University, School of Medicine, Xiamen University, Xiamen 361004, Fujian, China; 2 Institute of Gastrointestinal Oncology, Medical College of Xiamen University, Xiamen 361004, Fujian, China; 3 Xiamen Municipal Key Laboratory of Gastrointestinal Oncology, Xiamen 361004, Fujian, China

**Keywords:** mTOR, machine learning, cellular senescence, ming classification

## Abstract

Gastric cancer, as a tumor with poor prognosis, has been widely studied. Distinguishing the types of gastric cancer is helpful. Using the transcriptome data of gastric cancer in our study, relevant proteins of mTOR signaling pathway were screened to identify key genes by four machine learning models, and the models were validated in external datasets. Through correlation analysis, we explored the relationship between five key genes and immune cells and immunotherapy. By inducing cellular senescence in gastric cancer cells with bleomycin, we investigated changes in the expression levels of HRAS through western blot. By PCA clustering analysis, we used the five key genes for gastric cancer typing and explored differences in drug sensitivity and enrichment pathways between different clustering groups. We found that the SVM machine learning model was superior, and the five genes (PPARA, FNIP1, WNT5A, HRAS, HIF1A) were highly correlated with different immune cells in multiple databases. These five key genes have a significant impact on immunotherapy. Using the five genes for gastric cancer gene typing, four genes were expressed higher in group 1 and were more sensitive to drugs in group 2. These results suggest that subtype-specific markers can improve the treatment and provide precision drugs for gastric cancer patients.

## INTRODUCTION

Gastric cancer (GC) is a multifactorial disease, with many factors affecting its development, such as environmental and genetic factors [1]. Research shows that GC is the fourth leading cause of cancer deaths worldwide. After being diagnosed as late stage, the median survival rate of GC patients is less than 1 year [2]. GC is a highly invasive malignant tumor with significant heterogeneity, which has been widely studied by researchers [3]. Proper diet, early diagnosis, and personalized treatment can reduce the incidence of GC and improve patient prognosis [4]. GC is relatively rare in the young population, with a prevalence of no more than 10% [5].

Mechanistic target of rapamycin (mTOR) is a protein kinase regulating cell growth, survival, metabolism, and immunity [6, 7]. mTOR is usually assembled into several complexes such as mTOR complex 1/2 (mTORC1/2). mTOR catalyzes the phosphorylation of multiple targets. regulating protein synthesis, nutrients metabolism, growth factor signaling, cell growth, and migration [8]. Activation of mTOR promotes tumor growth and metastasis.

The mTOR signaling pathway has been extensively studied in GC. Studies have found that GLI can mediate mTOR-induced PD-L1 expression in GC cells [9]. Salidroside induces apoptosis and protective autophagy in GC cells through the PI3K/Akt/mTOR pathway [10]. CircNRIP1 promotes GC progression through the microRNA-149-5p/AKT1/mTOR pathway [11]. Cynaroside promotes cell proliferation, apoptosis, migration, and invasion through the MET/AKT/mTOR axis [12]. The mTOR signaling pathway is also closely related to aging. SHQA inhibits oxidative stress-induced aging and replication by inhibiting the Akt/mTOR pathway [13]. Erythromycin improves oxidative stress-induced cell aging through the PI3K-mTOR signaling pathway in chronic obstructive pulmonary disease [14]. Currently, there are many different perspectives on building machine learning models related to GC, but no model has been proposed from the perspective of the mTOR signaling pathway. We are the first to propose this model in GC research.

Our study used machine learning to screen for molecules involved in the mTOR signaling pathway in GC transcriptome data, resulting in 5 key genes that are predicted to be closely related to GC immune microenvironment and drug sensitivity.

## RESULTS

### Construction of mTOR signaling-related gene features in gastric cancer

**Figure 1:**
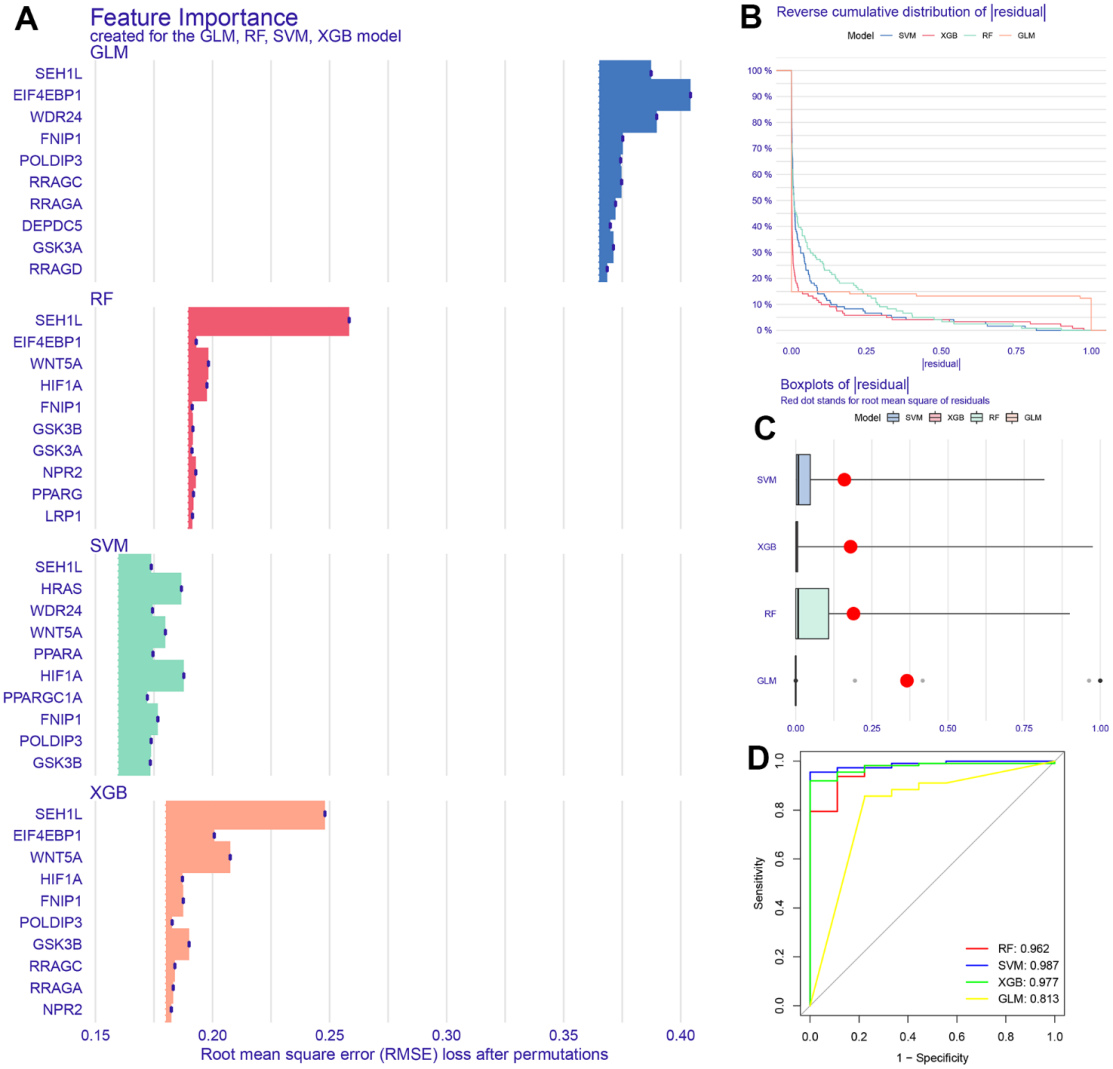
**Construction of machine learning model.** (**A**) Top 10 characteristic genes in 4 models. (**B**, **C**) Boxplots of residual and reverse cumulative distribution of residual. (**D**) The area under ROC curve of 4 models.

We identified a total of 47 key proteins involved in the mTOR signaling pathway. Using transcriptome data from TCGA-STAD, we obtained expression information for 32 genes. Machine learning models including Extreme Gradient Boosting (XGB), Random Forest (RF), Generalized linear model (GLM) and Support Vector Machine (SVM) were used to predict mTOR signaling-related features. Box plots of the top 10 most important genes in each model were generated (Figure 1A). Results from box plots of residuals and reverse cumulative distribution of residuals confirmed the superiority of the SVM model (Figure 1B, 1C). The area under the ROC curve for the SVM model was 0.987, while the performance of XGB, GLM, and RF machine learning techniques was inferior (ROC curve areas of 0.977, 0.813, and 0.962, respectively) (Figure 1D).

### Validation of the reliability of the SVM model in the GSE26942, GSE54129, GSE55696, and GSE66229 cohorts

**Figure 2:**
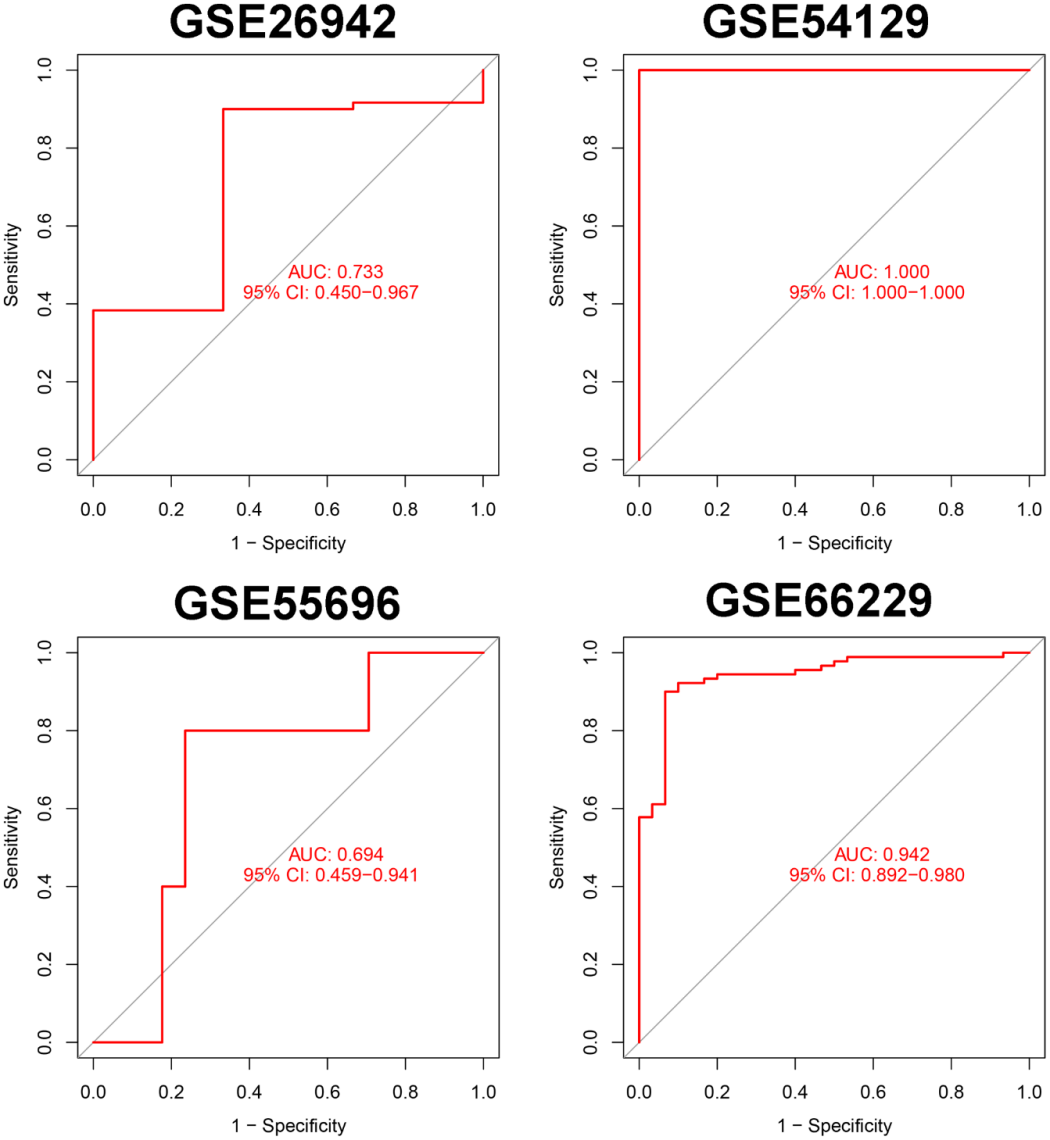
Verification of markers in 5 external data sets.

We selected the top 5 genes (PPARA, FNIP1, WNT5A, HRAS, and HIF1A) as feature genes in the SVM machine learning model and validated the model in the GSE26942, GSE54129, GSE55696, and GSE66229 cohorts. The AUC values after validation in the four external datasets were 0.733, 1.000, 0.694, and 0.942, respectively (Figure 2).

### Immune features related to key molecules in the mTOR signaling pathway in the model

**Figure 3:**
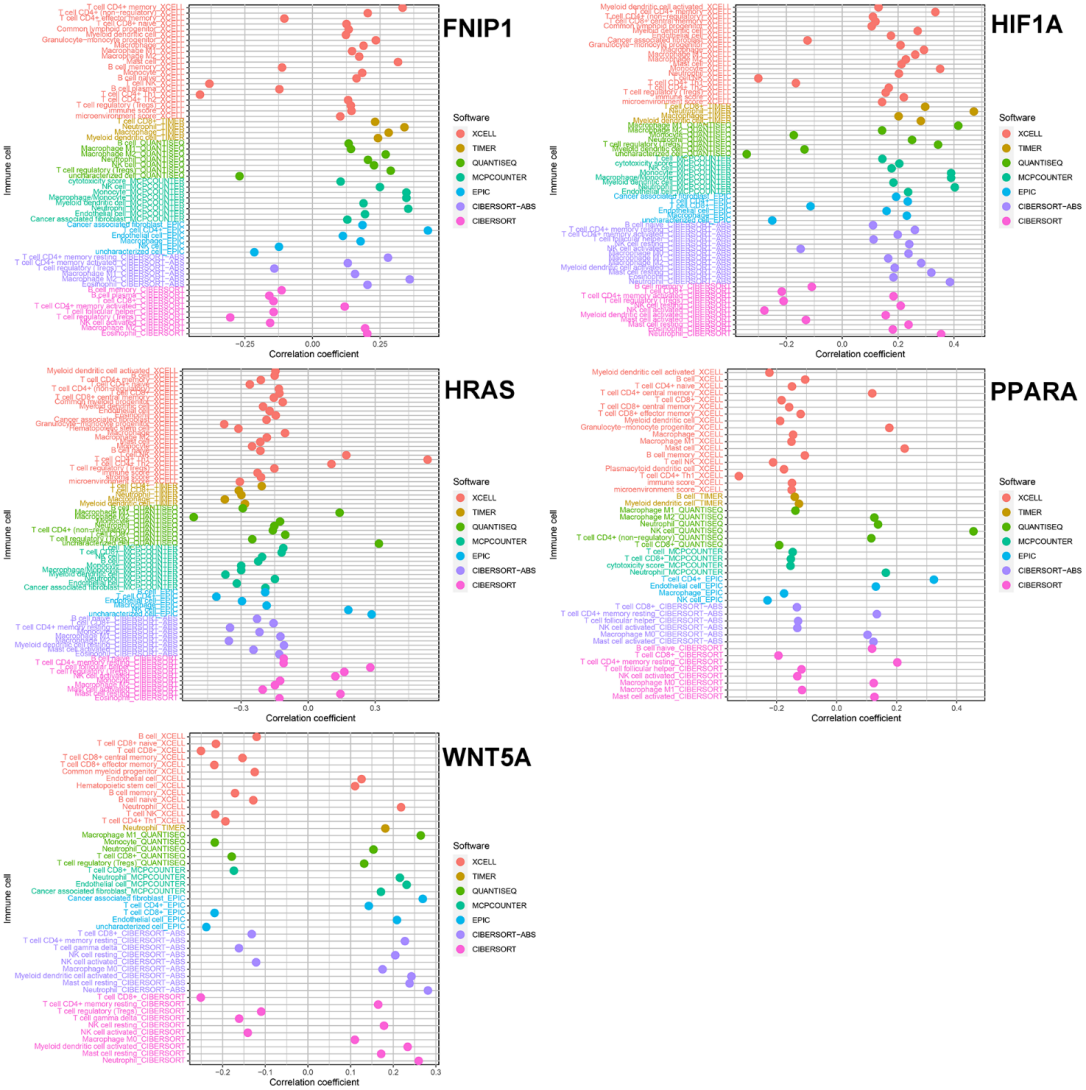
The relationship between five genes and immune cells.

We analyzed the correlation between these 5 genes and immune cells in 7 immune cell-related datasets (TIMER, CIBERSORT, CIBERSORT-ABS, QUANTISEQ, MCPCOUNTER, XCELL, and EPIC). In 4 of these datasets, the expression level of FNIP1 was positively correlated with M2 macrophages. The expression level of HIF1A was positively correlated with M1 and M2 macrophages, neutrophils, and myeloid dendritic cells, which has been confirmed in multiple databases. In 4 or more datasets, the expression level of HRAS was negatively correlated with B cells, M2 macrophages, monocytes, and CD8+ T cells. In 5 independent datasets, the expression level of PPARA was negatively correlated with CD8+ T cells. In 6 datasets, the expression level of WNT5A was positively correlated with neutrophils and negatively correlated with CD8+ T cells (Figure 3).

### Analysis of feature genes

**Figure 4:**
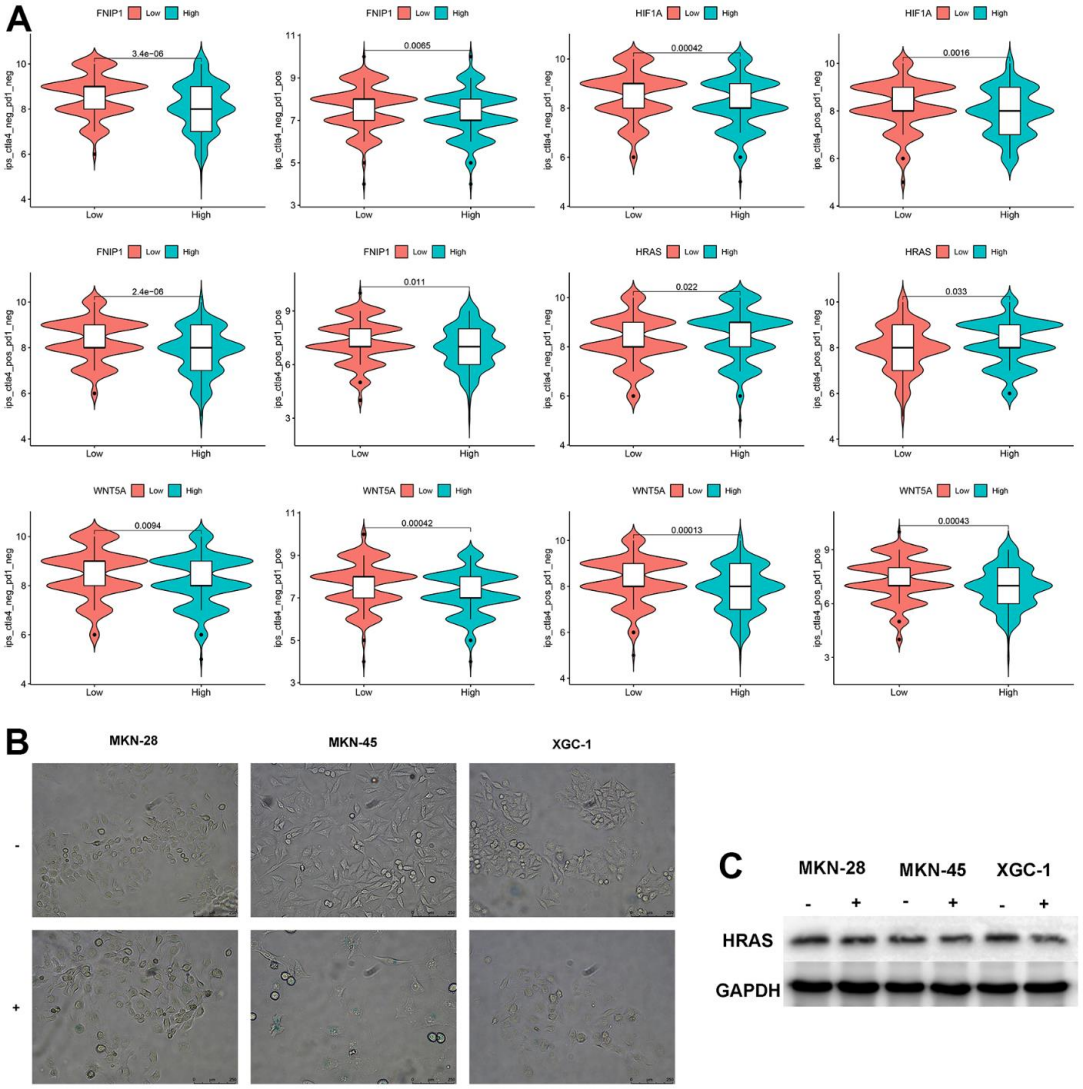
**Analysis of feature genes.** (**A**) The impact of characteristic genes on immunotherapy. (**B**) Bleomycin-induced cellular senescence (bar=250μm) (“-“represents the control group, “+” represents bleomycin induction). (**C**) Changes in HRAS expression levels after cellular senescence.

We analyzed the sensitivity of these 5 genes to immunotherapy and found that patients with low expression of FNIP1 and WNT5A were more sensitive to CTLA4, PD1, and combination therapy, while patients with low expression of HIF1A were more sensitive to CTLA4. Patients with high expression of HRAS were more sensitive to CTLA4. The expression level of PPARA showed no significant difference in response to CTLA4 and PD1 treatment (Figure 4A). Treatment with bleomycin induced cellular senescence in three types of GC cells, and the expression level of HRAS was significantly decreased in senescent cells (Figure 4B, 4C).

### Classification efficiency of feature genes

**Figure 5:**
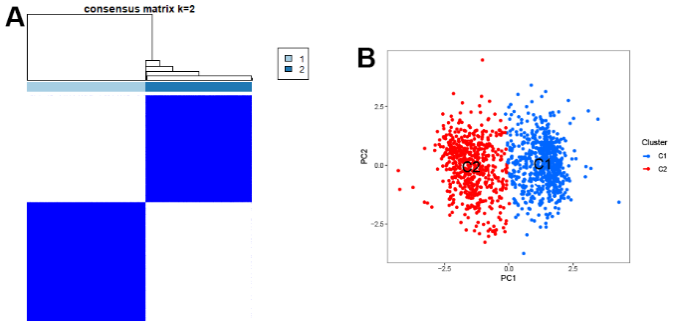
**Clustering of characteristic genes.** (**A**) Typing of characteristic genes for samples. (**B**) The scatter plot of PCA.

To confirm the representative role of these 5 genes in GC, we used these genes to classify a total of 1240 samples from three databases (TCGA, GSE84437, GSE26253). The results showed that these 5 genes could significantly divide the samples into two clusters (Figure 5A). The scatter plot of principal component analysis confirmed this result (Figure 5B), confirming the significant representativeness of the feature genes selected by the machine learning model.

### Comprehensive analysis between different clusters

**Figure 6:**
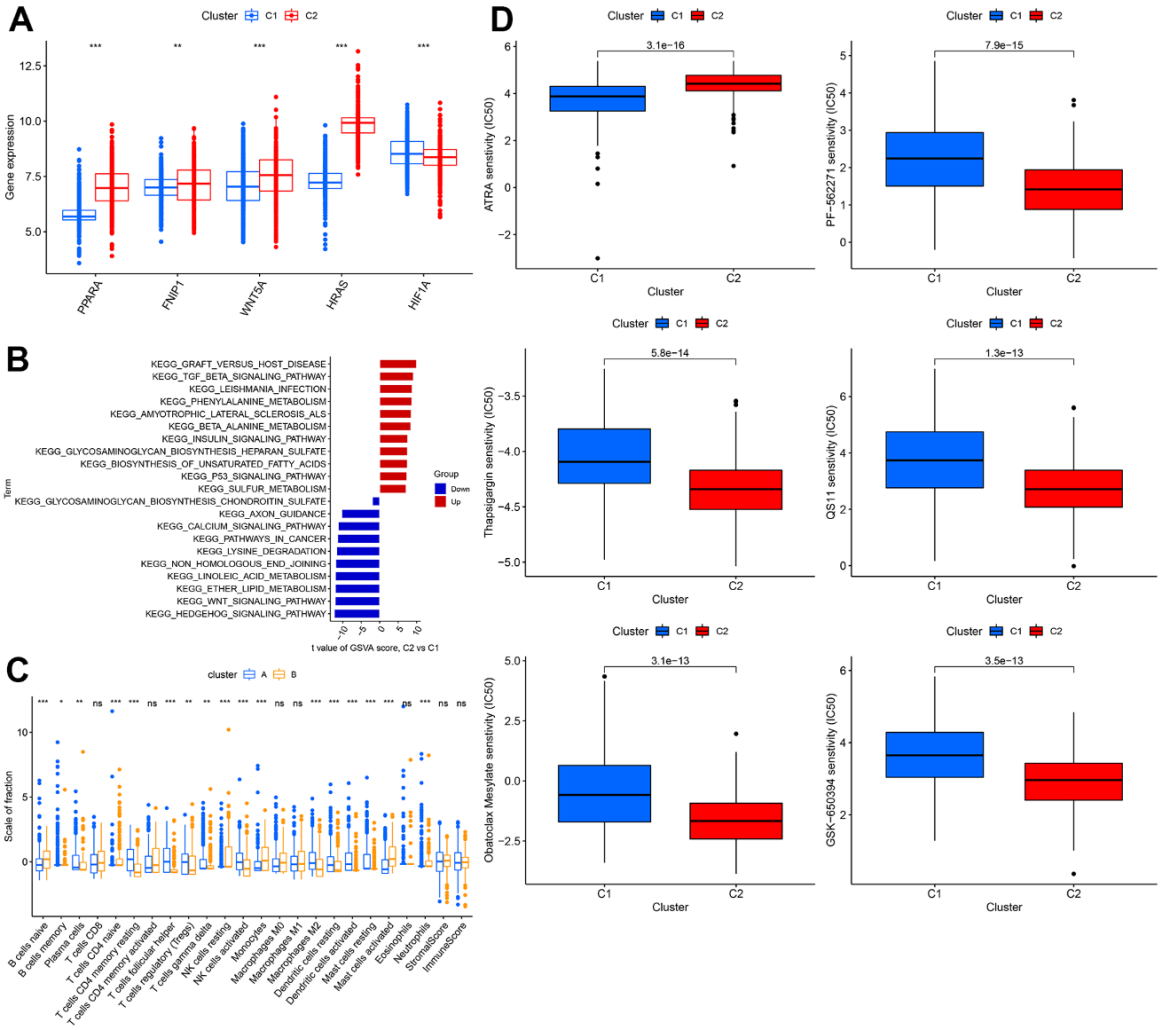
**Comprehensive analysis between different clusters.** (**A**) The expression level of WNT5A, FNIP1, PPARA, HRAS, HIF1A. (**B**) The GSVA analysis between 2 clusters. (**C**) Difference in distribution of immune cells in different cohorts. (**D**) The sensitivity to different drugs in different cohorts.

The expression levels of WNT5A, FNIP1, PPARA, and HRAS were significantly higher in cluster 1 than in cluster 2, while the expression of HIF1A was lower in cluster 1 than in cluster 2 (Figure 6A). Using GSVA analysis, we found that GRAFT_VERSUS_HOST_DISEASE was the most significant pathway in cluster 2, while HEDGEHOG_SIGNALING_PATHWAY was the most significant pathway in cluster 1 (Figure 6B). We estimated the immune cell content and immune scores for the 1240 samples. Most immune cells showed significant differences between the two clusters, but there were no significant differences in T cells CD8, T cells CD4 memory activated, Macrophages M0, Macrophages M1, Eosinophils, Neutrophils, StromalScore, ImmuneScore (Figure 6C). We also calculated the sensitivity of the two clusters to different drugs and presented the top 6 drugs with the most significant differences, showing that patients in cluster 2 were more sensitive to these 5 drugs than those in cluster 1 (Figure 6D).

## DISCUSSION

GC is a common malignant tumor in the digestive system [15]. Risk factors for GC include Helicobacter pylori infection, age, high salt intake, and insufficient fruit and vegetable consumption [16]. Although advanced diagnostic and treatment methods have reduced its incidence, GC remains the leading cause of cancer death in East Asia [17]. There is an urgent need for research and development of tumor markers. The heterogeneity of GC has driven the rapid development of tumor classification, from Ming classification, Borrmann classification, Lauren classification and WHO classification to various molecular classifications. The emergence of molecular classification has greatly promoted the progress of tumor treatment.

In our proposed classification, these five genes play an important role in the disease. In renal tumors, loss of FLCN-FNIP1/2 induces a non-classical interferon response, and FNIP1 and FNIP2 are crucial for FLCN’s tumor suppressor function [18]. FNIP1 plays an important role in regulating the specificity of skeletal muscle fiber type, anti-fatigue and susceptibility to muscular dystrophy. Calmodulin 2 promotes angiogenesis and metastasis of GC through STAT3/HIF-1A/VEGF-A [19]. Lactic acid promotes macrophage polarization in GC through MCT-HIF1α signaling, affecting the components of the tumor microenvironment [20]. Exosomes secreted by GC cells under hypoxic conditions promote the progression and metastasis of GC through the MiR-301a-3p/PHD3/HIF-1α positive feedback loop [21]. Erianin has been found to inhibit GC precancerous lesions by inhibiting the HRAS-PI3K-AKT signaling pathway [22]. HRAS overexpression predicts the response of gastrointestinal tumors to lenvatinib treatment [23]. Tipifarnib significantly improves disease control rate in HRAS-mutant salivary gland cancer patients [24]. PPARα is associated with histological types in GC [24]. Activation of PPARα can inhibit cell growth and induce apoptosis of GC cells [25]. Blocking PPARα can activate the IL-6/STAT3 pathway to prevent acetaminophen-induced liver injury [26]. IL-10 promotes Wnt5a-induced M2 polarization of tumor-associated macrophages, promoting the progression of colorectal cancer [27]. Gambogic acid can inhibit the progression of GC through the miR-26a-5p/Wnt5a signaling pathway [28]. HEF1 regulates tumor cell differentiation through the Wnt5a/β-catenin signaling pathway in GC [29].

The ultimate goal of GC classification is to better treat patients. We investigated the correlation between these 5 genes and immunotherapy. Currently, there are studies reporting that HIF-1α inhibitors are a promising method to enhance anti-tumor immunity and can synergize with anti-PD-1 to inhibit tumor growth *in vivo* [30]. The other 4 genes have less reported relevance to immunotherapy, and our study provides new ideas for immunotherapy in GC. Thapsigargin is widely known for inducing cell apoptosis [31], and samples in cluster 2 exhibit lower sensitivity to thapsigargin treatment, providing a new approach for GC treatment. The combination of Obatoclax Mesylate and nanoparticles has demonstrated strong anti-tumor effects in non-small cell lung cancer [32], making the combination with nanocarriers a promising research direction.

Overall, the newly constructed model has strong and stable sensitivity and specificity. However, there are limitations, and our analysis is based on public databases, and the exploration of molecular mechanisms is not deep enough. We will continue to focus on these five key genes and carry out subsequent experimental verification.

## MATERIALS AND METHODS

### Reagents

Bleomycin (mixture) was purchased commercially (CAS No: ST1450) from Beyotime Biotechnology, Shanghai, China. Primary antibodies used in the protein expression analysis were purchased from Abcam (HRAS, GAPDH).

### Cell culture and bleomycin treatment on cells

MKN28, MKN45 human gastric cells were purchased from BeNa Culture Collection (Suzhou, China). Infiltrative GC cells XGC-1 (Patent No.: CN103396994A) was obtained from the Zhongshan Hospital Xiamen University [33]. These cells were cultured in Roswell Park Memorial Institute (RPMI)-1640 medium (HyClone, Logan, UT, USA) supplemented with fetal bovine serum (FBS, 10%, HyClone) and streptomycin/penicillin (1%, Solarbio, Beijing, China) in a moist atmosphere with 5% CO2 at 37° C. Gastric cells were seeded with an initial cell density of 1 × 10^6^ cells per 100 mm cell culture plate and bleomycin were added to the cells and incubated for 48 h at 37° C in a humidified atmosphere under 5% CO2. Based on previous research [34], we established different dosage groups of 10μg/mL, 20μg/mL, 30μg/mL, 40μg/mL, and 60μg/mL. Finally, according to the staining results, MKN-45 was induced into senescence in the 10μg/mL group, MKN-28 in the 20μg/mL group, and XGC-1 in the 30μg/mL group.

### SA-β-galactosidase staining

Senescent cells were analyzed using senescence associated β-galactosidase (SA-β-gal) staining. Cells were grown in six-well plates, washed, fixed, and stained with the SA-β-gal cellular senescence assay kit (Beyotime Biotechnology). The sections were examined under a microscope.

### Western blotting

Total protein was separated using the RIPA lysis buffer (Solarbio). Protein extracts were separated by 10% SDS-polyacrylamide gel electrophoresis and then transferred to nitrocellulose membranes and was incubated with specific antibodies. The immunoblot was visualized through the enhanced chemiluminescence reagent kit (Beyotime Biotechnology, Shanghai, China).

### Data collection

Gene expression data and clinical information were obtained from The Cancer Genome Atlas [35] (https://portal.gdc.cancer.gov/) and the Gene Expression Omnibus (GEO) [36] (https://www.ncbi.nlm.nih.gov/geo/) (GSE84437, GSE26253, GSE26942, GSE54129, GSE55696, GSE66229). We followed the access rules of the TCGA and GEO databases during the data collection process. The data used in this study were from public databases and did not require approval from local ethics committees. Transform Fragments Per Kilobase of exon model per Million mapped fragments data of TCGA into Transcripts Per Kilobase of exon model per Million mapped reads data, then merges with data of GEO.

### Merging three datasets (GSE84437, GSE26253, TCGA)

Using the “limma” package, we converted the TCGA transcriptome data from FPKM format to TPM format similar to that of the GEO transcriptome data. The “sva” package was used to merge the transcriptome data from both TCGA and GEO databases.

### Development and validation of mTOR signaling pathway-related signature

Transcriptome data from 375 tumor tissues and 32 adjacent paired normal tissues were analyzed in R software. The mTOR signaling pathway-related proteins were statistically collected by consulting relevant literature. Extreme Gradient Boosting (XGB), Random Forest (RF), Generalized linear model (GLM) and Support Vector Machine (SVM) were analyzed using the “caret”, “DALEX”, “ggplot2”, “randomForest”, “kernlab”, “pROC”, and “xgboost” packages.

### Immune therapy analysis

Immune therapy data related to GC were downloaded from The Cancer Immunome Atlas (https://tcia.at/) database, and the differences in immune therapy between genes with different expression levels were analyzed using the “limma” package.

### Tumor microenvironment analysis

ESTIMATE was used to calculate the stromal, immune, and ESTIMATE scores. Finally, the CIBERSORT algorithm was used to analyze the infiltration differences of 22 immune cells among different samples.

### Exploration of different molecular clusters

The samples were clustered using the “ConsensusClusterPlus” and “limma” packages. The bar plot of GSVA was performed using the “reshape2”, “ggpubr”, “limma”, “GSEABase”, and “GSVA” packages. The box plot of drug sensitivity was performed using the “car”, “ridge”, “preprocessCore”, “genefilter”, and “sva” packages.

### Statistical analysis

Continuous variables were summarized through mean and standard deviations and compared through Wilcoxon test. Categorized variables were presented by frequency (n) and proportion (%), and then compared through ANOVA. All of which were performed through R software (Version 3.6.3, The R Foundation for Statistical Computing). P values were two-side and were considered to be statistically significant if they were lower than 0.05.
